# Systemic Mastocytosis as an Unconventional Cause of Variceal Bleeding: Think Outside the Box

**DOI:** 10.7759/cureus.629

**Published:** 2016-06-03

**Authors:** Moiz Ahmed, Mayurathan Kesavan, Basmah N Jilani, Saba Ahmed, Liliane Deeb

**Affiliations:** 1 Department of Internal Medicine, Staten Island University Hospital, Northwell Health, Staten Island, NY, USA; 2 Department of Gastroenterology, Staten Island University Hospital, Northwell Health, Staten Island, NY, USA; 3 Department of Internal Medicine, Nishtar Medical College, Multan, Pakistan

**Keywords:** infiltrative liver disease, variceal bleeding, systemic mastocytosis, non-cirrhotic portal hypertension

## Abstract

Systemic mastocytosis is a rare infiltrative disease involving the skin, bone marrow, digestive system, and liver. We report a case of a 59-year-old male who presented with a massive variceal bleed without any evidence of cirrhosis; however was later found to have severe perisinusoidal fibrosis with mast cells in portal tracts on liver biopsy and hypercellular mast cell infiltrated bone marrow. This rare case describes an out-of-the-ordinary reason of variceal bleeding with preserved liver function due to non-cirrhotic portal hypertension.

## Introduction

Systemic mastocytosis (SM) is a rare infiltrative disease caused by excessive mast cell proliferation and accumulation in the skin, bone marrow, digestive system, liver, and spleen. Common gastrointestinal symptoms include diarrhea, abdominal pain, and gastrointestinal (GI) bleeding predominantly due to peptic ulcer disease. Non-cirrhotic portal hypertension is peculiar in mastocytosis, and it could rarely present with variceal bleeding typically in the setting of preserved liver function. Microscopic infiltration of the hepatic portal spaces by abnormal mast cells leads to fibrosis, increased portal pressure, and varix formation in the absence of liver cirrhosis. We report an unusual case of systemic mastocytosis that was disclosed after a massive variceal bleeding.

## Case presentation

Note: Informed verbal consent was obtained from the patient.

**Figure 1:**
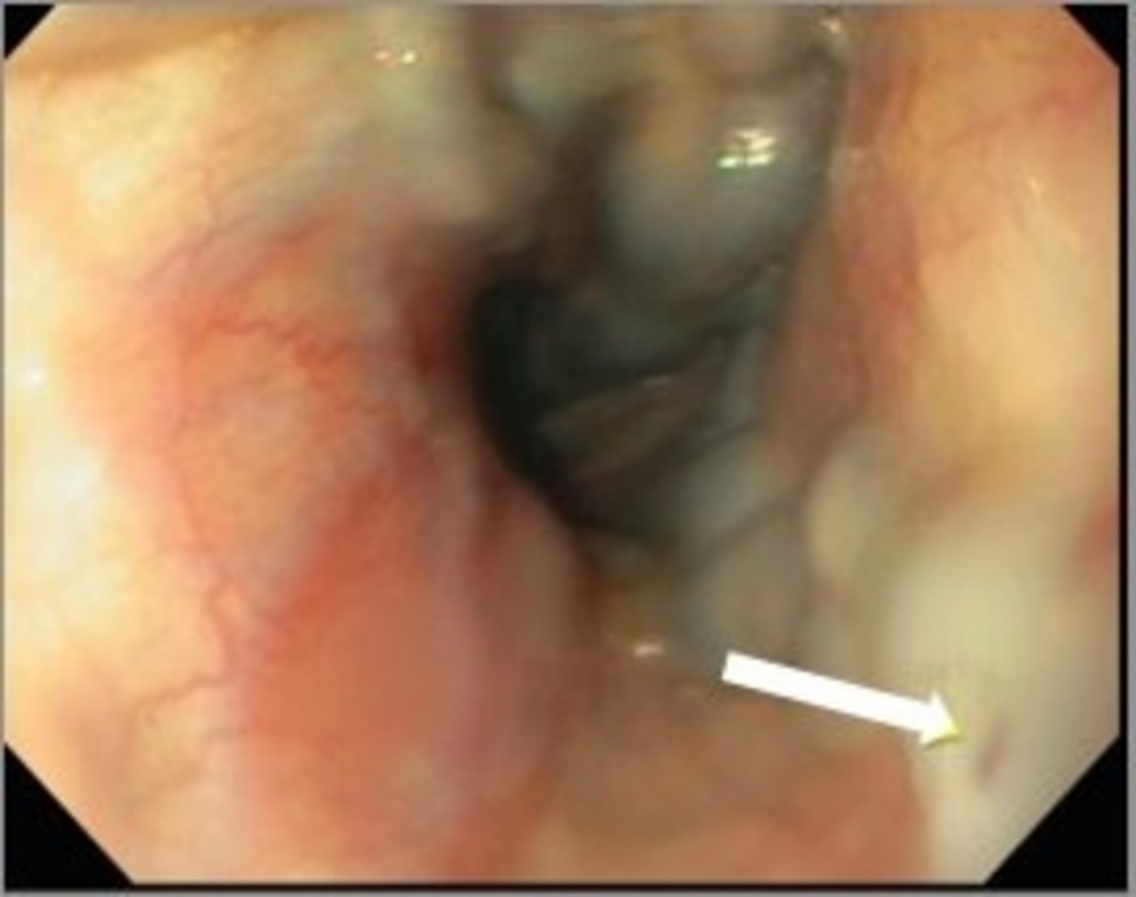
Upper endoscopy showing large esophageal varices with red wale sign (arrow)

**Figure 2:**
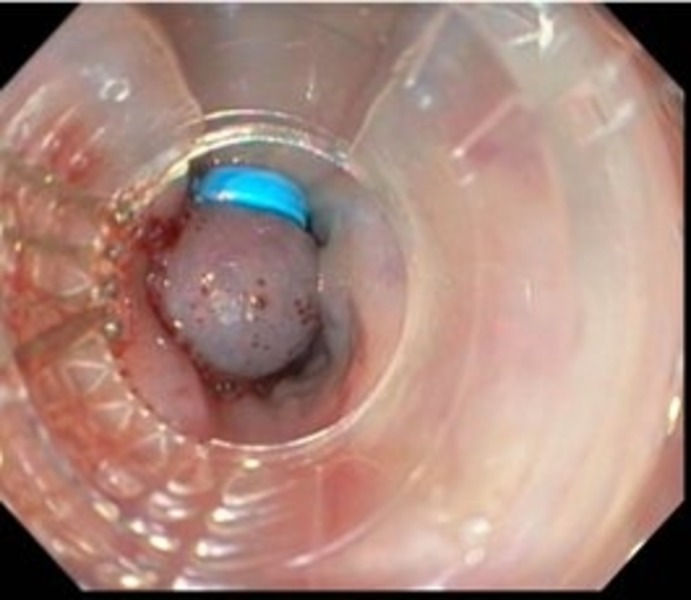
Upper endoscopy showing band ligated esophageal varices

A 59-year-old male with a history of smoldering multiple myeloma, coronary artery disease, and atrial fibrillation on coumadin presents to our emergency department with a three-day history of melenic stools, shortness of breath, and dizziness. He reported non-steroidal anti-inflammatory intake for muscle aches and denied hematemesis, abdominal pain, or weight loss. On presentation, he was tachycardic at 120-130 beats/minute and hypotensive with a blood pressure of 85/60 mm/Hg. His physical exam disclosed pale complexions and a soft, non-tender abdomen. A rectal examination confirmed the presence of black melenic stools, and a nasogastric lavage revealed bright red blood suggesting an upper gastrointestinal source of his bleeding. Laboratory tests revealed hemoglobin of 5.9 g/dL, blood urea nitrogen of 94 mg/dL, creatinine of 2.3 mg/dL, and a supratherapeutic INR of 15. Liver profile was unremarkable except for hypoalbuminemia of 2.3 mg/dL. Parenteral proton pump inhibitors were administered in a drip infusion. After achieving hemodynamic stability with fluid resuscitation, transfusion of two units of packed red blood cells, and correction of coagulopathy with four units of prothrombin complex concentrates (PCC), an emergent upper endoscopy (EGD) was performed. Large esophageal varices were noted uphill from distal to mid esophagus with ‘red wale’ signs of recent bleeding (Figure 1). Five ligation bands were successfully deployed with adequate decompression of the varices (Figure 2). Examination of the stomach also disclosed severe portal hypertensive gastropathy further suggesting the possibility of an undiagnosed portal hypertension in this patient. Octreotide infusion and IV antibiotics were administered thereafter.

**Figure 3:**
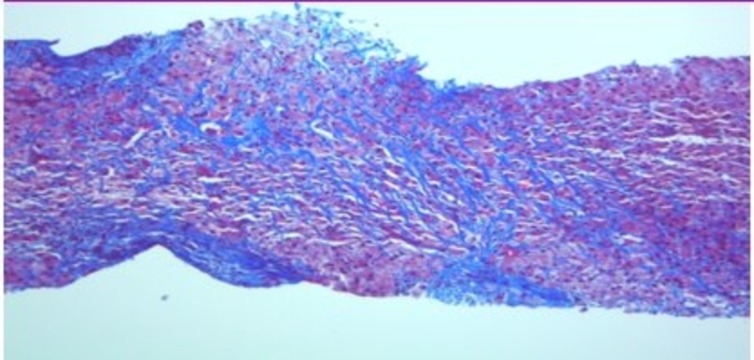
Liver biopsy (trichrome stain) revealing extensive portal and periportal fibrosis (in blue)

**Figure 4:**
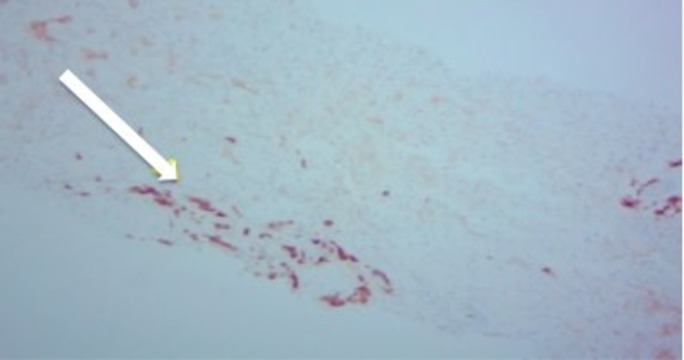
CD117 demonstrating abundant mast cells in portal tracts

Abdominal sonogram revealed normal liver parenchyma without any evidence of cirrhosis or ascites as well as patent hepatic and portal veins. Serologic tests for hepatitis A, B, C, HIV, markers of autoimmune hepatitis (AMA, ASMA, anti- LKM antibodies), ceruloplasmin, iron indices, ferritin, alpha-1-antitrypsin, and tumor markers were all unrevealing. Liver biopsy revealed severe perisinusoidal fibrosis with focal nodule formation (Figure 3). A CD117 immunostain disclosed abundant mast cells in portal tracts (Figure 4). Mast cell tryptase serum level was elevated: 104 ng/ml  (reference: 2 to 10 ng/ml), and bone marrow biopsy revealed hypercellular marrow infiltrated predominantly by mast cells, establishing the diagnosis of systemic mastocytosis. 

## Discussion

Systemic mastocytosis (SM) is a rare disease involving abnormal proliferation of mast cells into various organ systems. The gastrointestinal symptoms frequently seen are diarrhea and abdominal pain. Hepatomegaly with an infiltrative biochemical liver profile and splenomegaly could be other presenting signs as well. Elevated alkaline phosphatase is proved to be closely correlated with the severity of hepatic mast cell infiltration and fibrosis [1]. Unusually, liver size and biochemical profile could be intact despite extensive hepatic involvement as noted in our case; Those rare instances could pose a clinical challenge for the diagnosis and may lead to unwarranted delayed management.

Non-cirrhotic portal hypertension is a rare occurrence in SM which was first described by Capron et al., in 1978 [2]. Following that, five more cases were reported, of which two were associated with ascites [2-3]. Metabolic abnormalities such as hyperammonemia and hypoalbuminemia may accompany this condition while biochemical liver profile is generally preserved [4]. Portal fibrosis, as seen in our case, has been described in the literature in only 25 out of 182 cases [5]. It is due to accumulation of chronic inflammatory cells, namely clonally abnormal mast cells, resulting in fibrosis within the portal area while sparing the sinusoids [6]. The underlying mechanism is deposition of subendothelial collagen in the portal triad due to mast cell derived factors (e.g., tryptase, TNF- alfa, FGF, PDGF, chymase) [7-8] leading to periportal fibrosis [7], increased portal pressure, variceal formation, and bleeding [2, 9].

Recognition of mast cells with conventional staining of liver sections could be challenging due to the loss of cytoplasmic granules; however, they are easily seen with special stains like Giemsa and immunostain CD 117 [8]. Liver biopsy typically shows fibrous tissue entrapping portal tracts, spindled mast cells, and ductular reactions [10]. 

Treatment of SM is very difficult. Antihistamine therapy and cromolyn sodium can be used to decrease symptoms of gastric hypersecretion like dyspepsia, diarrhea, and malabsorption. Corticosteroids have been found useful in improving gastrointestinal symptoms and hepatic mast cell infiltration. Interferon and hydroxyurea are sometimes useful for complicated SM [1].

## Conclusions

The diagnosis of systemic mastocytosis becomes a challenge when it presents without superficial skin manifestations. It takes a thorough investigation to detect the source of a disease that has such a variable presentation. In conclusion, it is important for us clinicians to always consider SM as a cause of non-cirrhotic portal hypertension that could lead to variceal bleeding especially in the setting of preserved liver functions. This can be further confirmed by lab parameters like serum tryptase levels and immunohistochemical staining of liver biopsy. Heightened awareness of this unique and rare presentation of mastocytosis is advised to improve early recognition and avoid unnecessary investigation.
